# Long-Term Efficacy of Maintenance Therapy for Multiple Myeloma: A Quantitative Synthesis of 22 Randomized Controlled Trials

**DOI:** 10.3389/fphar.2018.00430

**Published:** 2018-04-30

**Authors:** Jie-Li Li, Guang-Yu Fan, Yu-Jie Liu, Zi-Hang Zeng, Jing-Juan Huang, Zong-Ming Yang, Xiang-Yu Meng

**Affiliations:** ^1^Department of Hematology, Zhongnan Hospital of Wuhan University, Wuhan, China; ^2^Center for Evidence-Based and Translational Medicine, Zhongnan Hospital of Wuhan University, Wuhan, China; ^3^Department of Evidence-Based Medicine and Clinical Epidemiology, Second Clinical College of Wuhan University, Wuhan, China

**Keywords:** multiple myeloma, maintenance, meta-analysis, network meta-analysis, trial-sequential analysis

## Abstract

We aimed to quantitatively synthesize data from randomized controlled trials (RCTs) concerning maintenance for multiple myeloma (MM). We searched electronic literature databases and conference proceedings to identify relevant RCTs. We selected eligible RCTs using predefined selection criteria. We conducted meta-analysis comparing maintenance containing new agents and conventional maintenance, and subgroup analysis by transplantation status and mainstay agent as well. We performed trial sequential analysis (TSA) to determine adequacy of sample size for overall and subgroup meta-analyses. We performed network meta-analysis (NMA) to compare and rank included regimens. A total of 22 RCTs involving 9,968 MM patients and 15 regimens were included, the overall quality of which was adequate. Significant heterogeneity was detected for progression-free survival (PFS) but not overall survival (OS). Meta-analyses showed that maintenance containing new agents significantly improved PFS but not OS [PFS: Hazard Ratio (HR) = 0.59, 95% Confidence Interval (CI) = 0.54 to 0.64; OS: HR = 0.93, 95% CI = 0.87 to 1.00], compared with controls. Subgroup analyses revealed lenalidomide (Len)-based therapies better than thalidomide-based ones (HR = 0.50 and 0.66, respectively; *P* = 0.001). NMA revealed that most of the maintenance regimens containing new agents were significantly better than simple observation in terms of PFS but not OS. Len single agent was the most effective, considering PFS and OS both. We concluded that conventional maintenance has very limited effect. Maintenance containing new agents is highly effective in improving PFS, but has very limited effect on OS. Maintenance with Len may have the largest survival benefits. Emerging strategies may further change the landscape of maintenance of MM.

## Introduction

Multiple myeloma (MM) is a common hematological malignancy that originates from plasma cells (Terpos and International Myeloma Society, 2017). Treatment strategies based on autologous stem cell transplantation (ASCT) and newly developed agents (e.g., Bor, Tha, and Len) have significantly improved the outcome of patients with MM (Chen J.F. et al., 2017; Zeng et al., 2017). However, due to inevitable disease progression and relapse, it remains incurable (Chen W.C. et al., 2017; Moreau and de Wit, 2017). To increase the long-term survival of MM patients, it has been widely investigated the efficacy of consolidation and maintenance therapies in enhancing and maintaining the response of initial treatment (Nathwani et al., 2016). Unlike consolidation which is used exclusively post-ASCT, maintenance can be applied to transplant-eligible and transplant-ineligible patients (McCarthy and Holstein, 2016). The major goal of post-induction and post-ASCT maintenance therapy is to improve MM patients’ prognosis by deepening and maintaining disease remission achieved with primary treatment. The interferon and glucocorticoids such as Pre and Dex, either alone or in doublet combination, were the first few agents tested and applied as MM maintenance in the 1990s. Since the first decade of the 21st century, novel anti-MM agents have been used in MM maintenance therapy (McCarthy and Holstein, 2016; Moreau and de Wit, 2017). The novel agents consist of two major categories, i.e., the immunomodulatory drugs (IMiDs) including Tha and Len, and proteasome inhibitors (PIs) such as Bor. The interferon, glucocorticoids, IMiDs and PIs exert anti-MM effects via different biological mechanisms (Nathwani et al., 2016). Although delay in relapse and prolonged progression-free survival (PFS) achieved by maintenance has been recorded in certain trials, disease recurrence and progression remain almost inevitable. Besides, inconsistency exists among the results of different studies, especially in terms of the benefits measured by overall survival (OS) (Sengsayadeth et al., 2017). Among dozens of randomized controlled trial (RCT) reports, only a few revealed significant benefits in OS resulted from MM maintenance, but the strength of effect was quite weak; others indicated that maintenance treatment had no significant impact on OS (Sengsayadeth et al., 2017). On the other hand, safety issues represent a major concern of MM maintenance therapy. Long-term use of anti-MM agents may cause minor to severe adverse drug reactions (ADRs) due to accumulated drug toxicity, such as impaired hematopoiesis, thrombosis, immune dysfunction, recurrent infection, gastrointestinal adverse effects, metabolic disorders, peripheral neuropathy, and osteonecrosis of femoral head (ONFH). These ADRs bring harm to patients’ health and quality of life, and may result in premature discontinuation of treatment due to intolerance to side effects. Moreover, long-term treatment may also become a huge economical burden to the patients and society. Therefore, careful consideration and evidence-based decision-making is required before a maintenance therapy is prescribed to any MM patients. To the best of our knowledge, although several systematic reviews and meta-analyses of RCTs have discussed the role of MM maintenance, the results are not conclusive; and there is no comprehensive comparison of multiple maintenance strategies from a quantitative perspective (Ye et al., 2013; Gao et al., 2014; Liu et al., 2015; Wang et al., 2016; McCarthy et al., 2017; Sun et al., 2017). Therefore, to provide updated and thoroughly summarized evidence for clinical decision-making regarding MM maintenance, we conducted this quantitative synthesis of available information from RCTs by performing meta-analysis, trial sequential analysis (TSA) and network meta-analysis (NMA), focusing on long-term efficacy.

## Materials and Methods

### Search Strategy

We searched the PubMed, Embase, Cochrane collaboration database, and the proceedings of major international conferences in hematology and oncology to identify relevant RCTs. We used the following search terms: “MM,” “maintenance therapy,” and “randomized.” We also manually screened the reference lists of included studies and previous meta-analyses to find additional studies. Only publications in English were considered. The last search was performed on 30 May.

### Study Selection

We used the following criteria for inclusion eligibility: (i) the study subjects were patients with symptomatic MM, either newly diagnosed or previously treated; (ii) the design was RCT; (iii) different maintenance treatments (including Obs or Pla) were compared, with at least one arm containing new agents (Tha, Len, and Bor); (iv) sufficient information was provided on PFS and/or OS. In case where data on PFS were not provided, data on event-free survival or time-to-progression were used as surrogates. Two investigators independently performed literature search and study selection. Any discrepancies were resolved by discussion with a third investigator.

### Data Extraction

Two investigators independently extracted the following data from the included studies: name of the first author, year of publication, number of patients, agents used for maintenance, and ASCT status. The hazard ratio (HR) and corresponding 95% confidence interval (95%CI) of survival endpoints were either extracted directly from study reports or calculated from Kaplan–Meier curves as described previously (Parmar et al., 1998; Tierney et al., 2007). Any discrepancies were resolved by discussion with a third investigator.

### Quality Assessment

Two investigators independently assessed the methodological quality of included RCTs using the Cochrane collaboration’s risk-of-bias assessment tool (Zeng et al., 2015). Any discrepancies were resolved by discussion with a third investigator. The Review manager 5.3 was used to record and present the results of quality assessment.

### Statistical Analysis

Inverse-variance weighted meta-analysis was performed for studies comparing maintenance therapies containing new agents and conventional controls (Obs without maintenance, Pla, or maintenance based-on IFN-α and/or corticosteroid such as Dex). Heterogeneity statistics were used to guide the choice of meta-analytic model. In brief, the fixed-effects model was used where the *I*^2^ ≤ 50% and *P*-value of *Q*-test > 0.1, otherwise the random-effects model was used (Higgins and Thompson, 2002). Subgroup analysis by ASCT status and mainstay agent (Tha or Len) was performed, and *Z*-test was performed to evaluate the difference between subtotals. Publication bias was evaluated using funnel plots and Begg’s test (Begg and Berlin, 1989; Begg and Mazumdar, 1994). TSA was performed for overall and subgroup meta-analyses to determine whether enough information accumulated for a definitive conclusion (Miladinovic et al., 2013). The a priori diversity-adjusted information size (APDIS) was used as the information measurement. The prespecified type I error was set as two-sided α = 0.05, and type II error as β = 20% (1 - β = 80% power). A conservative relative risk reduction (RRR) of 15% was used as described previously (Miladinovic et al., 2013). O’Brian-Fleming boundaries were used (DeMets and Lan, 1994). Frequentist NMA was performed to compare and rank all the included maintenance strategies (Salanti, 2012). Obs/Pla was used as the reference intervention. Consistency model would be used if no significant inconsistency was detected; otherwise inconsistency model would be used (Veroniki et al., 2013). The surface under the cumulative ranking curve (SUCRA) was calculated and used to rank all interventions, respectively, for PFS and OS (Salanti et al., 2011). Clustered ranking considering both PFS and OS was also performed (Chaimani et al., 2013). All statistical analyses were performed using the Stata 13.0 software. A *P*-value less than 0.05 and 95% CI not covering 1 was considered statistically significant.

## Results

### Included Studies and Their Characteristics

**FIGURE 1:**
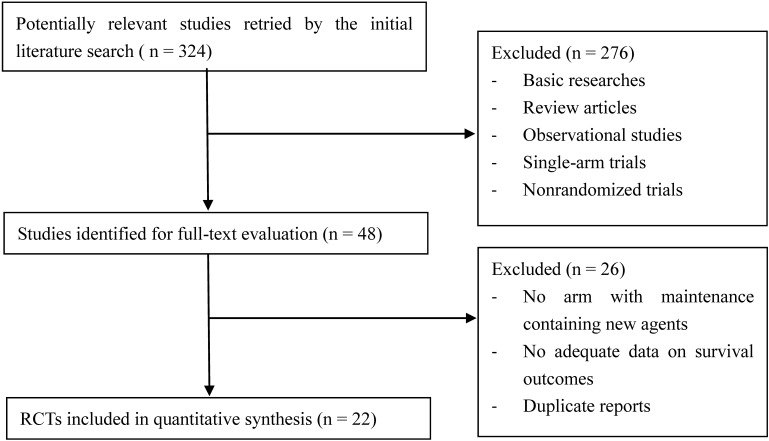
Flow diagram of literature search and study selection. Among 324 hits retrieved by initial search, 22 RCTs were finally identified eligible.

The flow diagram of literature search and study selection is shown in **Figure 1**. 324 potentially relevant studies were retrieved by the initial search. 276 studies were excluded after title and abstract screening. Another 26 studies were excluded after full-text evaluation. Finally, 22 RCTs (Attal et al., 2006, 2012; Barlogie et al., 2008; Palumbo et al., 2008, 2012, 2014; Rajkumar et al., 2008; Offidani et al., 2009; Lokhorst et al., 2010; Ludwig et al., 2010; Waage et al., 2010; Wijermans et al., 2010; Zonder et al., 2010; Maiolino et al., 2012; Mateos et al., 2012; McCarthy et al., 2012; Morgan et al., 2012; Stewart et al., 2013; Benboubker et al., 2014; Gay et al., 2015; Jackson et al., 2016; Rosinol et al., 2017) involving 9,968 MM patients and 15 different maintenance strategies were included in this quantitative synthesis.

The basic characteristics of the included RCTs were shown in **Table 1**. All the included studies contained two arms (Attal et al., 2006, 2012; Barlogie et al., 2008; Palumbo et al., 2008, 2012, 2014; Rajkumar et al., 2008; Offidani et al., 2009; Lokhorst et al., 2010; Ludwig et al., 2010; Waage et al., 2010; Wijermans et al., 2010; Zonder et al., 2010; Maiolino et al., 2012; Mateos et al., 2012; McCarthy et al., 2012; Morgan et al., 2012; Stewart et al., 2013; Benboubker et al., 2014; Gay et al., 2015; Jackson et al., 2016) except one three-arm trial (Rosinol et al., 2017). Three studies included both transplant-eligible and transplant-ineligible patients, and analyzed them separately (Morgan et al., 2012; Palumbo et al., 2014; Jackson et al., 2016). One study included both transplant-eligible and transplant-ineligible patients without separate analysis (Gay et al., 2015). Twenty studies compared maintenance containing new agents and conventional controls in terms of PFS, (Attal et al., 2006, 2012; Barlogie et al., 2008; Palumbo et al., 2008, 2012, 2014; Rajkumar et al., 2008; Offidani et al., 2009; Lokhorst et al., 2010; Ludwig et al., 2010; Waage et al., 2010; Wijermans et al., 2010; Zonder et al., 2010; Maiolino et al., 2012; McCarthy et al., 2012; Morgan et al., 2012; Stewart et al., 2013; Benboubker et al., 2014; Jackson et al., 2016; Rosinol et al., 2017) and 19 in terms of OS (Attal et al., 2006, 2012; Barlogie et al., 2008; Palumbo et al., 2008, 2012, 2014; Rajkumar et al., 2008; Offidani et al., 2009; Lokhorst et al., 2010; Ludwig et al., 2010; Waage et al., 2010; Wijermans et al., 2010; Zonder et al., 2010; Maiolino et al., 2012; McCarthy et al., 2012; Morgan et al., 2012; Stewart et al., 2013; Benboubker et al., 2014; Rosinol et al., 2017). Two studies investigated regimens containing Bor, (Mateos et al., 2012; Rosinol et al., 2017) 8 studies investigated Len-based regimens, (Zonder et al., 2010; Attal et al., 2012; McCarthy et al., 2012; Palumbo et al., 2012, 2014; Benboubker et al., 2014; Gay et al., 2015; Jackson et al., 2016) and 14 studies investigated Tha-based regimens (Attal et al., 2006; Barlogie et al., 2008; Palumbo et al., 2008; Rajkumar et al., 2008; Offidani et al., 2009; Lokhorst et al., 2010; Ludwig et al., 2010; Waage et al., 2010; Wijermans et al., 2010; Maiolino et al., 2012; Mateos et al., 2012; Morgan et al., 2012; Stewart et al., 2013; Rosinol et al., 2017).

**Table 1:** Included RCTs and basic characteristics.

Author	Year of publication	Number of patients	Maintenance	ASCT
Attal et al.	2006	597	Tha+Pam vs. Obs/Pla	Yes
Barlogie et al.	2008	668	Tha+IFN-α+Dex vs. IFN-α+Dex	Yes
Palumbo et al.	2008	331	Tha vs. Obs/Pla	No
Rajkumar et al.	2008	466	Tha+Dex vs. Dex	No
Offidani et al.	2009	103	Tha+IFN-α vs. IFN-α+Dex	No
Lokhorst et al.	2010	536	Tha vs. IFN-α	Yes
Ludwig et al.	2010	128	Tha+IFN-α vs. IFN-α	No
Waage et al.	2010	357	Tha vs. Obs/Pla	No
Wijermans et al.	2010	333	Tha vs. Obs/Pla	No
Zonder et al.	2010	192	Len+Dex vs. Dex	No
Attal et al.	2012	614	Len vs. Obs/Pla	Yes
Maiolino et al.	2012	108	Tha+Dex vs. Dex	Yes
Mateos et al.	2012	178	Bor+Tha vs. Bor+Pre	No
McCarthy et al.	2012	460	Len vs. Obs/Pla	Yes
Morgan et al. (a)	2012	492	Tha vs. Obs/Pla	Yes
Morgan et al. (b)	2012	326	Tha vs. Obs/Pla	No
Palumbo et al.	2012	305	Len vs. Obs/Pla	No
Stewart et al.	2013	332	Tha+Pre vs. Obs/Pla	Yes
Benboubker et al.	2014	1076	Len+Dex vs. Obs/Pla	No
Palumbo et al. (a)	2014	116	Len vs. Obs/Pla	Yes
Palumbo et al. (b)	2014	115	Len vs. Obs/Pla	No
Gay et al.	2015	223	Len+Pre vs. Len	Mixed
Jackson et al. (a)	2016	828	Len vs. Obs/Pla	Yes
Jackson et al. (b)	2016	722	Len vs. Obs/Pla	No
Rosinol et al.	2017	390	Bor+Tha vs. Tha vs. IFN-α	Yes

### Risk-of-Bias Assessment

**FIGURE 2:**
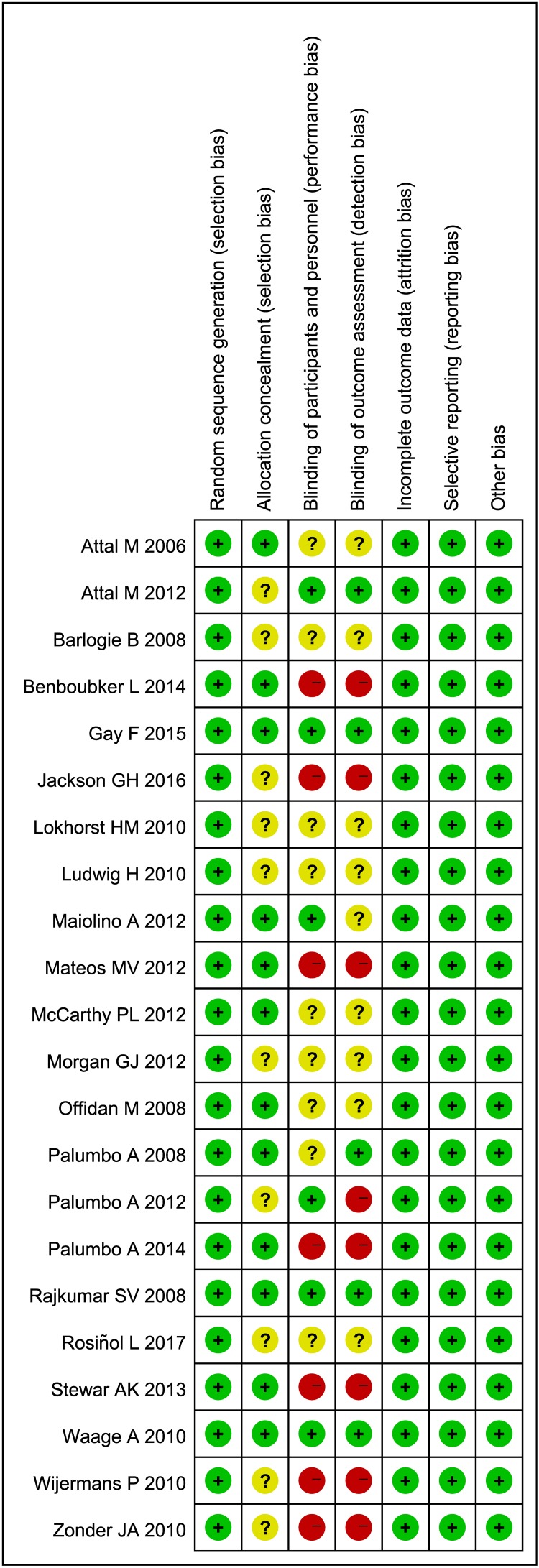
Summary of risk-of-bias assessment results for included studies. Included studies are of adequate methodological quality.

The summary of risk-of-bias assessment is shown in **Figure 2**. Regarding random sequence generation, incomplete outcome data, selective reporting and other bias, all the included RCTs were rated “low risk.” 10 studies were rated “unclear risk” in terms of allocation concealment. 9 studies were rated “unclear risk” and 7 studies were rated “high risk” in terms of blinding of participants and personnel. 9 studies were rated “unclear risk” and 8 studies were rated “high risk” in terms of blinding of outcome assessment. The overall quality of the included studies is adequate.

### Meta-Analysis

**FIGURE 3:**
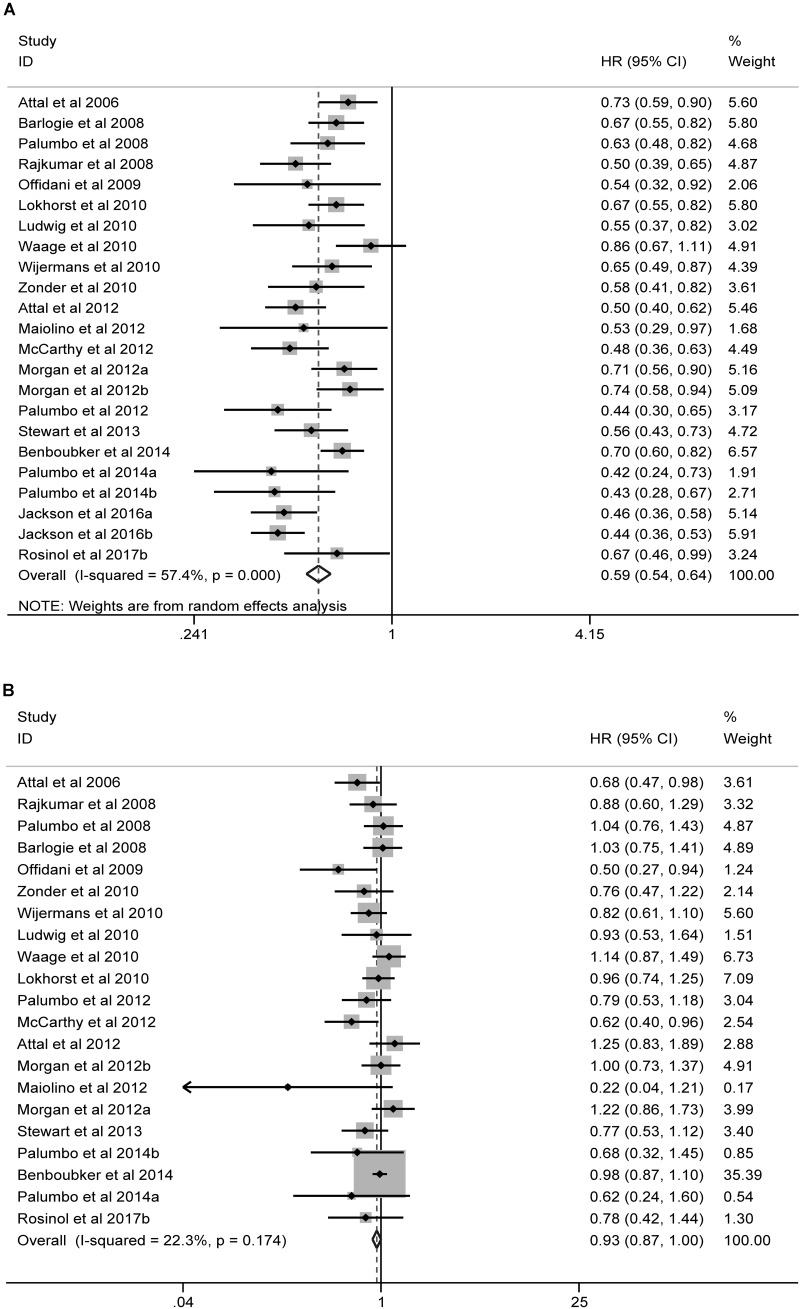
Forest plots of meta-analysis of RCTs comparing maintenance containing new agents and conventional maintenance. Significant and non-significant beneficial effects were found associated with maintenance using new agents, respectively, in terms of PFS **(A)** and OS **(B)**.

The forest plots of meta-analysis comparing maintenance containing new agents and conventional controls are illustrated in **Figure 3**. Significant heterogeneity was detected for PFS (*I*^2^ = 57.4%; Cochran *Q*-test: *P* < 0.001), and the pooled results by random-effects model showed that maintenance containing new agents significantly improved the PFS (HR = 0.59, 95% CI = 0.54 to 0.64; *P* < 0.001), as compared with controls. In contrast, no significant heterogeneity (*I*^2^ = 22.3%; Cochran *Q*-test: *P* = 0.174) was found for OS, and the improvement in OS was minimal and marginally non-significant (HR = 0.93, 95% CI = 0.87 to 1.00; *P* = 0.055), as compared with controls. Similar results were observed in subgroup analyses by ASCT status or mainstay agents (**Supplementary Figure S1**). No significant difference between subtotals was observed except that Len-based therapies were associated with significantly larger benefits in PFS as compared with Tha-based therapies (HR = 0.50 and 0.66, respectively, for Len-based therapies and Tha-based therapies; *Z*-test: *P* = 0.001). Funnel plots for PFS was symmetric (**Supplementary Figure S2A**; Egger’s test: *P* = 0.148) but OS asymmetric (**Supplementary Figure S2B**; Egger’s test: *P* = 0.03), which indicated potential publication bias for OS.

### Trial Sequential Analysis (TSA)

The results of TSAs were shown in **Supplementary Figure S3**. For PFS, the cumulative Z-curves crossed the monitoring boundaries in overall and subgroup TSAs, indicating firm evidence of effect (true positive). In contrast, for OS, the cumulative Z-curves did not cross the monitoring boundaries with patients more than the required information size included, which indicated lack of effect (true negative), in all TSAs except the one focusing on the Len subgroup in which the required APDIS was not reached, which indicated absence of evidence of effects (false negative).

### Network Meta-Analysis (NMA)

A total of 14 maintenance strategies were included in the NMA. The network plots were shown in **Supplementary Figure S4**. Since no significant inconsistency detected (*P* > 0.05), the consistency model was used for the NMAs. The NMA results were presented in **Table 2**, with Obs/Pla as reference. For PFS, most of the maintenance regimens containing new agents were significantly better than Obs without active treatment. In contrast, for OS, no significant difference was noted between Obs and anyone of the other regimens. The SUCRA plots were illustrated in **Supplementary Figure S5**. Maintenance with Len and Pre was ranked first in terms of PFS, and maintenance with Tha and Pam was ranked first in terms of OS. Averaged mean rank and clustered ranking both showed that the maintenance with Len single agent was the most effective strategy, considering PFS and OS both, as presented in **Supplementary Figure S6**.

**Table 2:** Network meta-analysis results, with observation/placebo as the common reference.

Maintenance	PFS		OS	
	HR	95%CrI	HR	95%CrI
Bor+Pre	0.90	(0.52, 1.56)	1.24	(0.47, 3.24)
Bor+Tha	0.61	(0.43, 0.88)	0.85	(0.47, 1.55)
Dex	1.21	(0.83, 1.77)	1.29	(0.75, 2.22)
IFN-α	1.01	(0.82, 1.24)	1.08	(0.79, 1.48)
IFN-α+Dex	1.02	(0.51, 2.05)	2.01	(0.79, 5.09)
Len	0.46	(0.42, 0.51)	0.82	(0.65, 1.04)
Len+Dex	0.70	(0.60, 0.82)	0.98	(0.80, 1.20)
Len+Pre	0.39	(0.27, 0.56)	1.26	(0.61, 2.59)
Tha	0.72	(0.64, 0.81)	1.03	(0.88, 1.20)
Tha+Dex	0.61	(0.39, 0.95)	1.05	(0.53, 2.07)
Tha+IFN-α	0.56	(0.35, 0.87)	1.00	(0.51, 1.96)
Tha+IFN-α+Dex	0.68	(0.33, 1.41)	2.07	(0.76, 5.60)
Tha+Pam	0.73	(0.59, 0.90)	0.68	(0.46, 1.02)
Tha+Pre	0.56	(0.43, 0.73)	0.77	(0.51, 1.16)

## Discussion

The main objective of maintenance treatment in MM patients is to prolong the duration of response and survival (Sengsayadeth et al., 2017). In previous studies, the effect of conventional maintenance using corticosteroid and/or IFN-α is highly limited, and better efficacy performance has been observed with maintenance based on new agents. Although it has been widely tested in RCTs and further summarized in subsequent meta-analyses, the effect of maintenance therapies in improving patients’ survival remains undetermined (Ye et al., 2013; Gao et al., 2014; Liu et al., 2015; Wang et al., 2016; Sun et al., 2017). The present quantitative synthesis for the first time evaluated the conclusiveness of meta-analysis results by performing TSA, and compared all available regimens by performing NMA.

The first agent ever used for MM maintenance is the interferon. The estimated potential mechanisms of interferon’s anti-MM effects as maintenance include the prolongation of cell cycle and overall generation time, G0/G1 blockage, inhibition of proliferation and self-renewal of MM cells, and immunomodulating effects. Although a few pilot studies reported that maintenance using interferon might increase duration of response and bring prognostic benefits to MM patients, recent reports revealed negative findings (Barlogie et al., 2008; Offidani et al., 2009; Lokhorst et al., 2010; Ludwig et al., 2010; Sengsayadeth et al., 2017). The reported adverse effects (AEs) of interferon maintenance mainly include influenza-like syndrome, hematologic toxic reactions and impaired renal and/or hepatic function. The AEs can be serious and may persist even the dosage reduced. Given its toxic effects, limited patient compliance and unclear benefits in terms of long-term outcome, interferon is generally not used alone for MM maintenance but in combination of other agents (Barlogie et al., 2008; Offidani et al., 2009; Lokhorst et al., 2010; Ludwig et al., 2010). Glucocorticoids, such as Pre and Dex, have demonstrated efficacy in myeloma no matter used alone or in combination, the mechanism of which mainly relies on suppressing cytokine production essential for MM. The major side effects of long-term use of steroids include immune dysfunction, recurrent infection, metabolic disorders, gastrointestinal tract mucosa damage and ONFH. Like interferon, the steroids are always used in combination with other agents instead of alone, due to considerable side effects, low degree of compliance and unclear long-term benefits (Rajkumar et al., 2008; Zonder et al., 2010; Maiolino et al., 2012). Since the first few years of 2000s, new anti-myeloma agents, i.e., the IMiDs and PIs, have been applied for post-induction or post-ASCT maintenance in MM patients. These drugs showed evident beneficial effects in trials involving patients with refractory and/or relapsed MM, among which the Tha, Len, and Bor are the most widely used in clinical practice for MM maintenance, as single agent or in combined regimens (Chen J.F. et al., 2017; Sengsayadeth et al., 2017; Terpos and International Myeloma Society, 2017; Zeng et al., 2017). Tha and Len are IMiDs. Their primary anti-MM effects include inhibition of cell proliferation, induction of apoptosis, anti-angiogenesis and immunomodulating effects. Attal et al. (2006) for the first time reported significantly improved PFS and OS among patients treated with Tha maintenance. However, Tha may result in multiple serious ADRs, such as abnormal hematopoiesis, deep vein thrombosis, pulmonary thromboembolism, gastrointestinal AEs, skin rash and polyneuritis, particularly during long-term use. With persistent uncertainty regarding OS benefits, limited adherence and high discontinuation rate due to its poor adverse-effect profile, Tha maintenance is not always considered with priority (Sengsayadeth et al., 2017). Len is a more effective and less toxic derivate of Tha, in both settings of induction chemotherapy and maintenance. Although it has been expected that Len as maintenance may show stronger long-term benefits, inconsistent findings are observed among multiple trials, particularly in terms of OS. Len has similar adverse-effect profile with Tha, but shows more potent inhibitory effect on hematopoiesis and potential risk of second primary malignancy (Zonder et al., 2010; Attal et al., 2012; McCarthy et al., 2012). Len-based regimens are currently considered first-line choices for MM maintenance. Due to uncertainties about the optimal duration of Len maintenance, most patients are recommend to take the drug till disease progression, but it may result in severe ADRs due to accumulated toxicity, premature discontinuation due to intolerable side reactions, and huge economic burden due to long-term drug use (Zonder et al., 2010; Attal et al., 2012; McCarthy et al., 2012). The efficacy and safety of maintenance regimens containing Bor has also been investigated. According to Mateos et al. (2012) although patients treated by TV (Tha + Bor) maintenance tended to have better PFS and OS than TP (Tha + Pre) maintenance, the difference was not statistically significant. According to Rosinol et al. (2017), adding Bor to Tha will bring significant PFS and OS benefits. However, the combination largely increased the risk for multiple ADRs including hematologic toxic effects and peripheral neuropathy (Mateos et al., 2012; Rosinol et al., 2017). Therefore, the TV regimen may only be considered for specific subjects who tolerates its poor safety profile.

Based on our meta-analyses and TSAs, it is very likely that maintenance therapies containing new agents can significantly improve patients’ PFS, compared with simple Obs or conventional maintenance, regardless of ASCT status and type of mainstay agents. However, maintenance therapies containing new agents have no beneficial effects on OS, regardless of ASCT status. Tha-based maintenance therapies bring no benefits in OS, and false-negativity may be present regarding the effect of Len-based maintenance on OS. The NMAs revealed similar findings. The conventional maintenance is no better than simple Obs, in terms of PFS and OS both, regardless of the agents used. Compared with Obs or Pla, the majority of maintenance therapies containing new agents significantly improved PFS, but none was associated with significant improvement in OS. Very interestingly, an obvious inconsistency between PFS and OS was observed in meta-analyses, TSAs and NMAs. As additional exploration, we performed a weighted (by sample size) least square linear regression to investigate the relationship between the logarithmic point estimates of HRs of PFS and OS, and we found that the correlation between PFS and OS were non-significant (β = 0.6150, *P* = 0.0534; **Supplementary Figure S8**). Based on this finding, it is suspected that the PFS may not be a good surrogate of OS in MM maintenance trials.

Currently, there are several ongoing trials investigating new maintenance strategies with new generations of proteasome inhibitors (e.g., ixazomib and carfilzomib), histone deacetylase inhibitors (e.g., vorinostat and panobinostat) and monoclonal antibodies (e.g., elotuzumab and daratumumab), including several RCTs. An updated NMA can be performed when their final results become available. The basic information of ongoing RCTs and the network map of the updated NMA are shown in **Supplementary Figure S7**. These investigational maintenance treatments with promising agents may bring solid benefits in OS and further change the landscape of maintenance for MM.

The present study has certain limitations. First, significant heterogeneity was detected for PFS, which persisted in subgroupanalyses by ASCT status and mainstay drug. Interestingly, we noted that in the subgroup analyses by mainstay drug, there wasno significant heterogeneity among studies identified that the study by Benboubker et al. (2014) was the most influential among studies comparing Len-based regimens and controls (*I*^2^ = 61%). After excluding this study from meta-analysis, the *I*^2^ dramatically decreased from 61 to 0%. Therefore, this study is probably an important source of heterogeneity. In addition, we speculate that the difference in induction regimen or ASCT protocols, risk-of-bias profile and duration of follow-up may also be potential sources of heterogeneity among trials. Second, potential risk of publication bias was indicated regarding OS meta-analysis. The trim-and-fill adjusted meta-analysis result is similar to the original one (original: HR = 0.93, 95% CI = 0.87 to 1.00; adjusted: HR = 0.96, 95% CI = 0.87 to 1.07), indicating that the influence of potential risk of publication bias may be minor. Third, certain comparisons (e.g., Dex vs. Obs/Pla) in NMA were primarily based on indirect evidence, which had limited precision and power.

To summarize, the following conclusions are made: (i) conventional maintenance has very limited effect; (ii) maintenance containing new agents is highly effective in improving PFS, but has very limited effect on OS; (iii) maintenance with Len may have the largest survival benefits. In clinical practice, potential benefits, risks of adverse events, costs and patient’s preference should be considered and balanced for the choice of maintenance strategy. Emerging strategies may further change the landscape of maintenance of MM.

## Author Contributions

X-YM designed and conceptualized the study. J-LL, G-YF, Y-JL, Z-HZ, J-JH, Z-MY, and X-YM participated in the literature search. J-LL and X-YM performed the data analysis. G-YF, Y-JL, and Z-HZ participated in quality assessment of the included studies. All authors participated in the reporting.

## Conflict of Interest Statement

The authors declare that the research was conducted in the absence of any commercial or financial relationships that could be construed as a potential conflict of interest.

## Supplementary Material

FIGURE S1 Forest plots of meta-analysis of randomized controlled trials (RCTs) comparing maintenance containing new agents and conventional maintenance, regarding progression-free survival (PFS) by autologous stem cell transplantation (ASCT) **(A)** and mainstay drug **(B)**, and overall survival (OS) by ASCT **(C)**, and mainstay drug **(D).**

FIGURE S2 Funnel plots of meta-analysis of RCTs comparing maintenance containing new agents and conventional maintenance, regarding PFS **(A)** and OS **(B)**.

FIGURE S3 Trial sequential analysis (TSA) results. The left column for PFS and right for OS. Each row from top to bottom corresponds to the overall analysis, and analyses for the subgroup of ASCT trials, subgroup of no ASCT trials, subgroup of thalidomide-based therapies and subgroup of lenalidomide-based therapies, respectively.

FIGURE S4 Network map of included maintenance strategies for PFS **(A)** and OS **(B)**. The size of node is weighted by the number of direct comparisons, and the size of edge weighted by the precision which is measured by reciprocal of the sampling variance.

FIGURE S5 Surface under the cumulative ranking curve (SUCRA) plots of NMA for PFS **(A)** and OS **(B)**.

FIGURE S6 Ranks accounting for both OS and PFS, based-on clustered ranking **(A)** and averaged mean rank **(B)**.

FIGURE S7 The summary of the ongoing RCTs and predicted network map of the updated NMA when their final results become available.

FIGURE S8 Ordinary and weighted (by sample size) least-square linear regression for the relationship between PFS and OS in MM maintenance trials. Logarithmic HRs of OS and PFS reported in 21 included studies with 24 independent datasets were investigated. Though ordinary regression revealed significant positive correlation between PFS and OS (β = 0.9637, *P* = 0.0123), weighted regression with more precision indicated non-significant association (β = 0.6150, *P* = 0.0534).

## Abbreviations

Bor: bortezomib
Dex: dexamethasone
IFN-α: interferon-α
Len: lenalidomide
Obs: observation
Pam: pamidronate
Pla: placebo
Pre: prednisone
Tha: thalidomide
