# Supplementary figures and images for: Long-Term Efficacy of Maintenance Therapy for Multiple Myeloma: A Quantitative Synthesis of 22 Randomized Controlled Trials
Source: Front Pharmacol. 2018 Apr 30;9:430. doi: 10.3389/fphar.2018.00430 (PMC5936780; doi:10.3389/fphar.2018.00430)

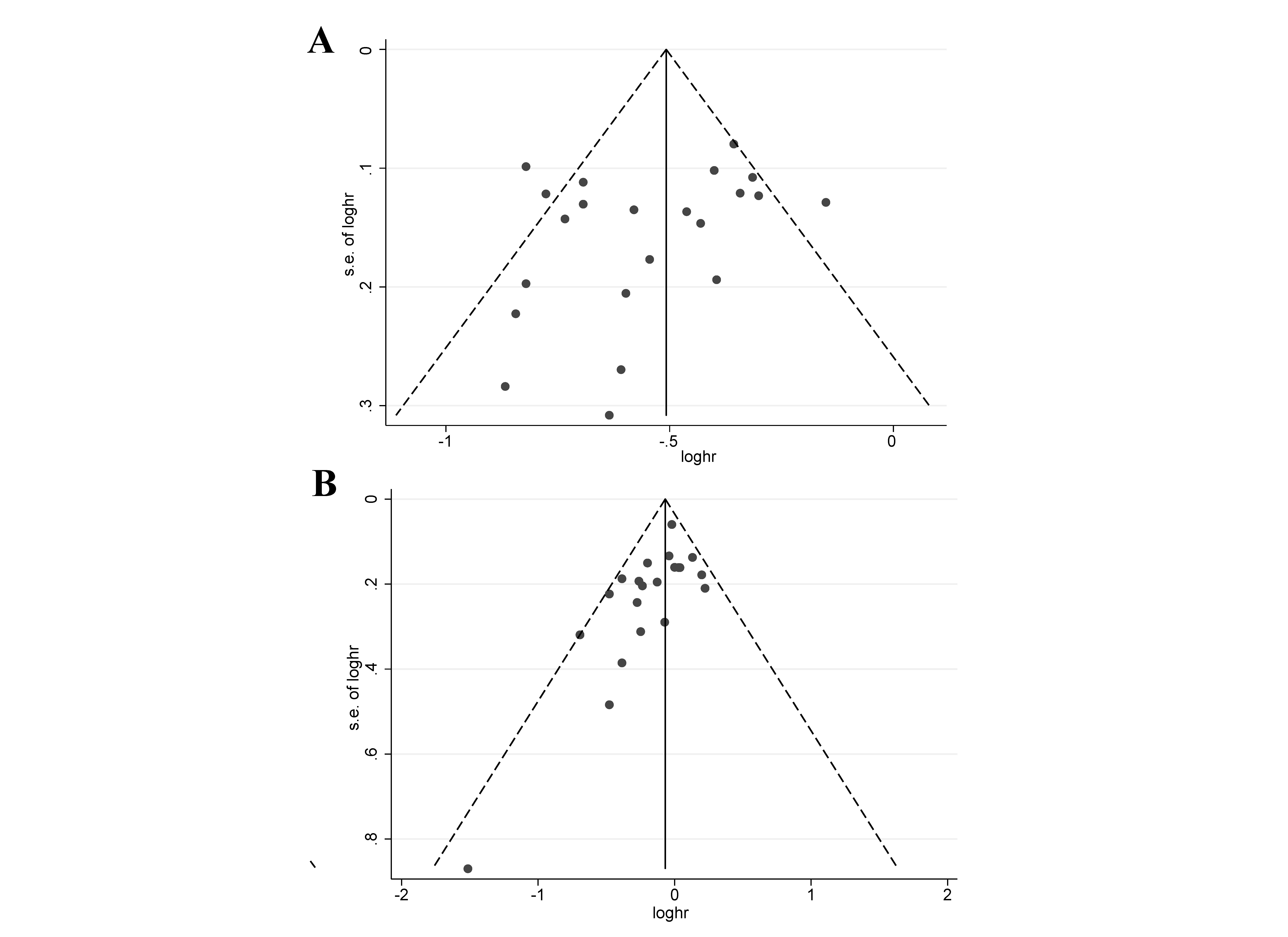

Supplement: FIGURE S2 — Funnel plots of meta-analysis of RCTs comparing maintenance containing new agents and conventional maintenance, regarding PFS (A) and OS (B). [file Image_2.JPEG]

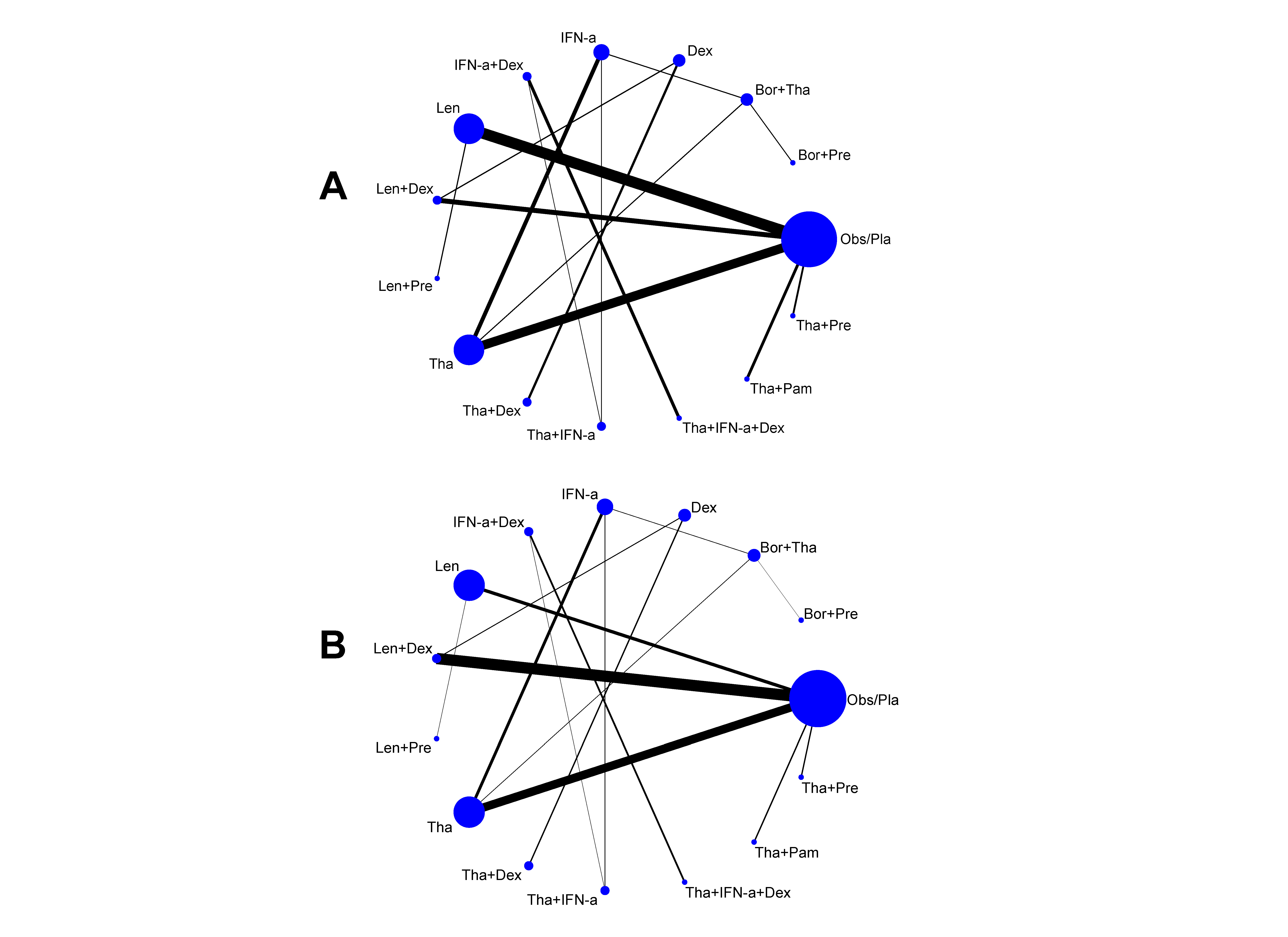

Supplement: FIGURE S4 — Network map of included maintenance strategies for PFS (A) and OS (B). The size of node is weighted by the number of direct comparisons, and the size of edge weighted by the precision which is measured by reciprocal of the sampling variance. [file Image_4.JPEG]

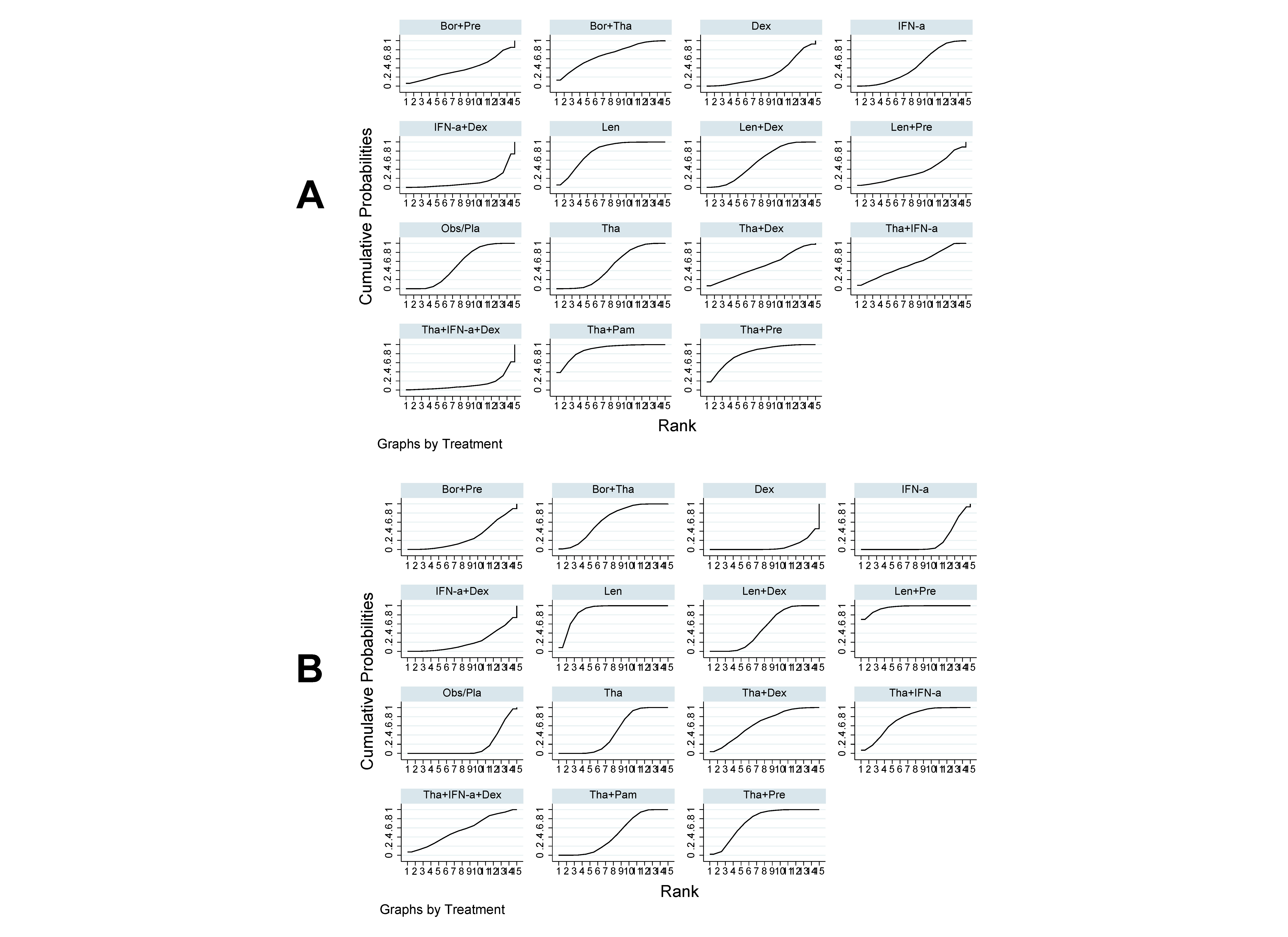

Supplement: FIGURE S5 — Surface under the cumulative ranking curve (SUCRA) plots of NMA for PFS (A) and OS (B). [file Image_5.JPEG]

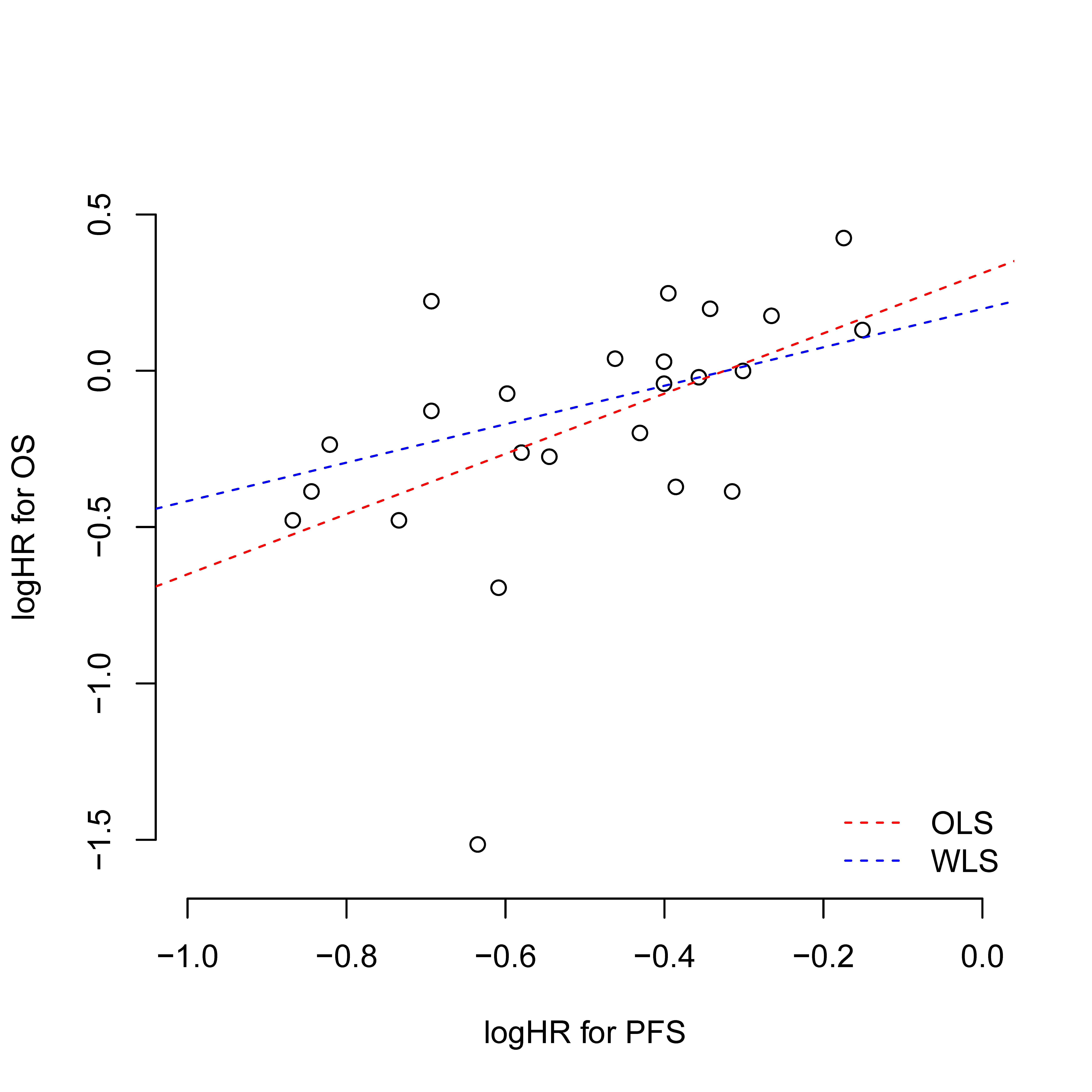

Supplement: FIGURE S8 — Ordinary and weighted (by sample size) least-square linear regression for the relationship between PFS and OS in MM maintenance trials. Logarithmic HRs of OS and PFS reported in 21 included studies with 24 independent datasets were investigated. Though ordinary regression revealed significant positive correlation between PFS and OS (β = 0.9637, P = 0.0123), weighted regression with more precision indicated non-significant association (β = 0.6150, P = 0.0534). [file Image_8.TIFF]
